# Supplementary figures and images for: Differentially Expressed miRNA of Prostate Cancer Compared with Benign Prostatic Hyperplasia Tissues: VAMP Associated Protein B Could Be Used for New Targets and Biomarkers of Prostate Cancer
Source: Biomedicines. 2025 Nov 28;13(12):2922. doi: 10.3390/biomedicines13122922 (PMC12730212; doi:10.3390/biomedicines13122922)

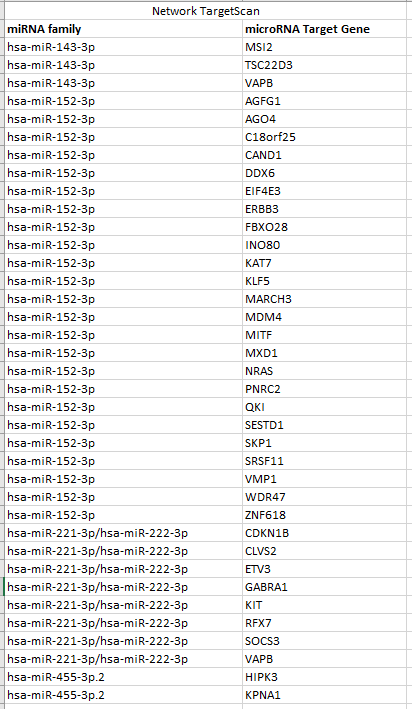

Supplement: Supplementary file 1 [file biomedicines-13-02922-s001.zip › biomedicines-3955007-Figure S1.png]
